# Simultaneous free-breathing T1, T2, and T1ρ mapping for myocardial fibrosis detection in non-ischemic cardiomyopathy: A comparative study with conventional techniques

**DOI:** 10.1016/j.jocmr.2025.101982

**Published:** 2025-11-03

**Authors:** Yali Wu, Xianling Qian, Kai Liu, Zhenfeng Lyu, Shiyu Wang, Yinyin Chen, Ling Chen, Zhuolin Liu, Lin Tian, Hang Jin, Haikun Qi, Mengsu Zeng

**Affiliations:** a Shanghai Institute of Medical Imaging, Shanghai, China; b Department of Radiology, Zhongshan Hospital, Fudan University, Shanghai, China; c School of Biomedical Engineering & State Key Laboratory of Advanced Medical Materials and Devices, ShanghaiTech University, Shanghai, China; d Shanghai Clinical Research and Trial Center, Shanghai, China; e Circle Cardiovascular Imaging Inc, Calgary, Alberta, Canada

**Keywords:** Multiparametric mapping, Free-breathing, Hypertrophic cardiomyopathy, Dilated cardiomyopathy

## Abstract

**Background:**

Quantitative myocardial mapping is critical for tissue characterization in non-ischemic cardiomyopathy (NICM). However, conventional techniques require separate breath-hold acquisitions, prolonging scan time and impairing co-registration. This study aimed to assess the feasibility and diagnostic performance of a novel free-breathing multimap (FBmultimap) sequence enabling simultaneous T1, T2, and T1ρ mapping in a single acquisition.

**Methods:**

Onehundred-nine participants were prospectively enrolled, including 48 with hypertrophic cardiomyopathy (HCM), 28 with dilated cardiomyopathy (DCM), and 33 healthy controls. All underwent cardiac MRI with both FBmultimap and conventional mapping sequences (modified Look-Locker inversion recovery (MOLLI) T1, T2-prepared balanced steady-state free precession (bSSFP), and T1ρ-prepared bSSFP). Image quality was assessed using subjective (four-point Likert scale) and objective (edge sharpness) methods. Myocardial relaxation times were analyzed in the following two subgroups: (1) HCM and DCM vs. controls, and (2) late gadolinium enhancement (LGE)-positive and LGE-negative patients vs. controls. Combined diagnostic indices (T1 + T1ρ) were derived using logistic regression. Diagnostic performance was evaluated using receiver operating characteristic analysis across the following six models: FBmultimap (T1 + T1ρ), FBmultimap T1, FBmultimap T1ρ, conventional (T1 + T1ρ), MOLLI T1, and T1ρ-prepared bSSFP, with area under the curve (AUC) calculated.

**Results:**

FBmultimap significantly reduced total scan time for T1 + T2 + T1ρ mapping to 66 ± 6 s, compared with 195 ± 10 s using conventional methods (*p*<0.001), while maintaining comparable image quality (all *p*>0.05). T1 and T1ρ values measured by FBmultimap were significantly elevated in HCM and DCM groups compared to controls, regardless of LGE status (all *p*<0.05), whereas T2 values showed no significant differences. FBmultimap (T1 + T1ρ) achieved higher AUCs for distinguishing LGE-positive (0.904) and LGE-negative (0.859) patients from controls than FBmultimap T1 (0.877 and 0.829), FBmultimap T1ρ (0.608 and 0.764), MOLLI T1 (0.770 and 0.671), T1ρ-prepared bSSFP (0.734 and 0.778), and the conventional (T1 + T1ρ) model (0.801 and 0.819).

**Conclusion:**

FBmultimap enables rapid, co-registered, free-breathing mapping of myocardial T1, T2, and T1ρ with high reproducibility and improved diagnostic performance over conventional single-parameter methods. It holds promise as a clinically applicable tool for myocardial fibrosis detection, risk stratification, and longitudinal monitoring in patients with HCM and DCM.

## Graphical abstract

**Figure:**
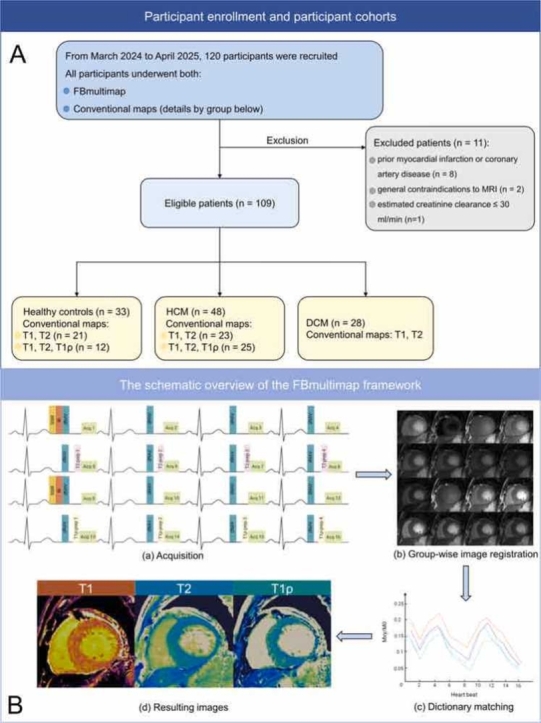

## 1 Introduction

Diffuse myocardial fibrosis is a major contributor to adverse remodeling and increased arrhythmic risk in non-ischemic cardiomyopathy (NICM) [1], [2]. Quantitative relaxation mapping provides a non-contrast approach for tissue characterization as follows: native T1 reflects extracellular volume expansion, T2 indicates edema or inflammation, and T1ρ is sensitive to macromolecular content, serving as a potential endogenous marker of diffuse fibrosis in both hypertrophic and dilated cardiomyopathy (HCM and DCM) [3], [4]. However, each parameter probes only a specific part of the myocardial tissue composition, and conventional techniques require separate acquisitions [5], [6]. Simultaneous mapping of T1, T2, and T1ρ may provide a more comprehensive and efficient assessment of diffuse myocardial abnormalities.

Conventional single-parameter mapping sequences—such as MOdified Look Locker Inversion recovery (MOLLI) T1 or T2-prepared balanced steady state free precession (bSSFP)—require separate breath-holds for each map, thereby prolonging scan times, introducing inter-scan misregistration and posing challenges for patients with limited breath-hold capacity [5], [7], [8]. Emerging techniques, including joint T1/T2 mapping, cardiac magnetic resonance imaging fingerprinting (cMRF), multimapping, and multitasking MRI, enable the simultaneous quantification of multiple parameters within a single acquisition [9], [10], [11], [12], [13], [14], [15]. Although these methods have shown promise in phantom, healthy volunteers, and small patients’ cohorts, their clinical validation remains limited. Free-breathing multimap (FBmultimap) is a free-breathing technique that simultaneously acquires spatially co-registered T1, T2, and T1ρ maps using B1-insensitive magnetization preparation and dictionary-based matching. It eliminates the need for breath-holds and idle recovery periods. Preliminary work has demonstrated technical feasibility in phantom and healthy volunteers, but its clinical utility in cardiomyopathy has not been established [16].

This study aimed to assess the feasibility and diagnostic performance of FBmultimap by comparing it with conventional MOLLI T1, T2-prepared bSSFP, and T1ρ-prepared bSSFP sequences in terms of acquisition time, image quality, and diagnostic accuracy in patients with HCM, DCM, and healthy controls. We hypothesized that FBmultimap would allow efficient and comprehensive myocardial tissue characterization by simultaneously acquiring co-registered T1, T2, and T1ρ maps under free-breathing conditions, while achieving diagnostic performance comparable to conventional mapping techniques with substantially reduced scan time.

## 2 Materials and methods

### 2.1 Study participants

**Fig. 1:**
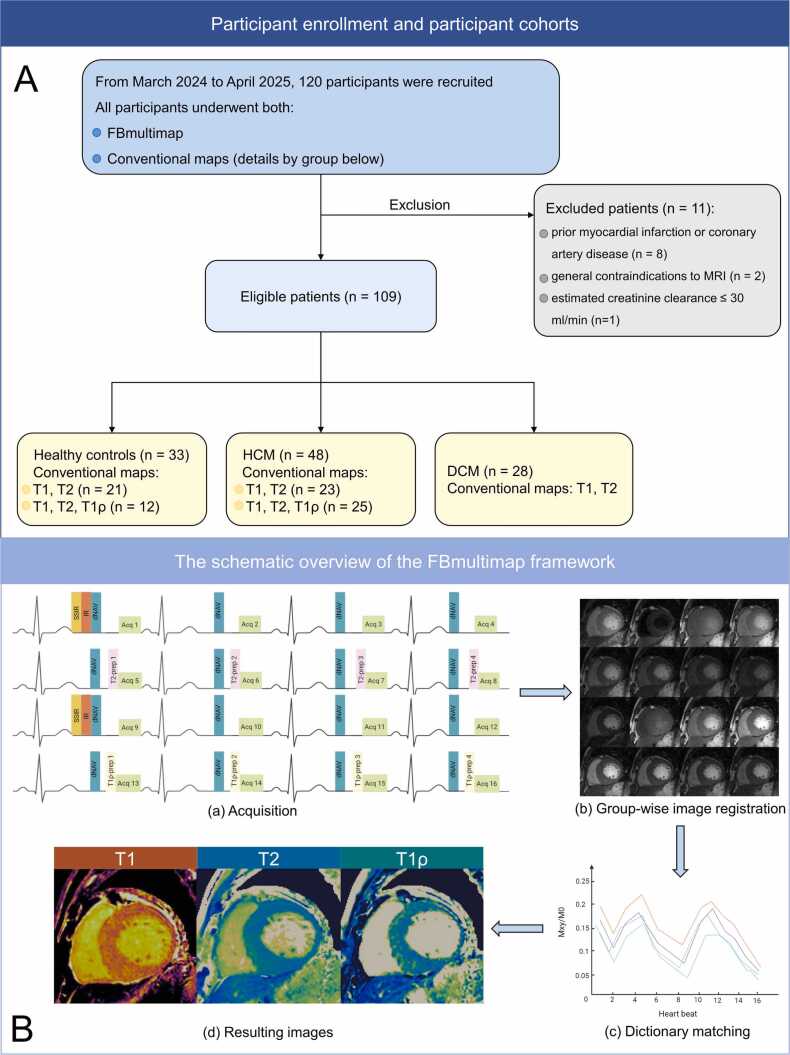
The study flowchart (**A**) and schematic overview of the FBmultimap framework (**B**). (**A**) A total of 109 participants were finally enrolled and categorized into three cohorts: healthy controls (*n* = 33), patients with (HCM, *n* = 48), and patients with (DCM, *n* = 28). (**B**) Schematic representation of the FBmultimap acquisition and reconstruction pipeline: (**a**) Sixteen cardiac cycles were acquired using IR, T2-prep, and T1ρ-prep pulses to encode T1, T2, and T1ρ sensitivity. SSIR and dNAV were used for prospective respiratory motion compensation. (**b**) In-plane motion was corrected using group-wise image registration. (**c**) Voxel-wise signal evolutions were matched to a Bloch-simulated dictionary accounting for T1, T2, T1ρ, and B1 variations. (**d**) Final parametric maps of T1, T2, and T1ρ were generated from dictionary matching. *FBmultimap* free-breathing multimap, *HCM* hypertrophic cardiomyopathy, *DCM* dilated cardiomyopathy, *IR* inversion recovery, *T2-prep* T2-prepared, *T1ρ-prep* T1ρ-prepared, *SSIR* slice-selective inversion recovery, *dNAV* diaphragm navigator

This prospective study was approved by the institutional ethics committee (Approval No. B2023–371), and written informed consent was obtained from all participants. From March 2024 to April 2025, we prospectively enrolled 48 patients with HCM and 28 with DCM according to the diagnostic criteria of the European Society of Cardiology (ESC) [17]. HCM was defined as a non-dilated, hypertrophied left ventricle (LV) with a maximal wall thickness ≥15 mm on cardiac MRI, or ≥13 mm in adults with an affected first-degree relative, in the absence of other causes of hypertrophy (e.g., hypertension, valvular disease, athlete’s heart). Diagnosis was further supported by typical cardiovascular magnetic resonance (CMR) findings, including asymmetric septal or apical hypertrophy and typical late gadolinium enhancement (LGE) patterns. DCM was diagnosed based on LV dilatation and systolic dysfunction, in the absence of abnormal loading conditions, coronary artery disease, valvular disease, or other identifiable causes. All diagnoses were determined by consensus following independent review by two experienced cardiac radiologists. Exclusion criteria included (a) prior myocardial infarction or coronary artery disease; (b) general contraindications to MRI (e.g., claustrophobia, pregnancy, non-compatible pacemaker/defibrillator, or intraocular/intracranial metallic materials); (c) known allergy to gadolinium-based contrast agents; (d) estimated creatinine clearance ≤ 30 mL/min; and (e) inability to provide informed consent. Additionally, 33 healthy individuals were prospectively recruited during the same period as research controls. All had normal electrocardiograms, no cardiovascular risk factors or known diseases and were not taking any medications (Fig. 1**A**). Normal reference ranges for mapping values were calculated from this cohort as mean ± 2 standard deviation (SD) [7].

### 2.2 CMR acquisition protocol

All examinations were performed on a 3.0-T clinical MR system (uMR 880; United Imaging, Shanghai, China) equipped with a 24-channel body coil. Three short-axis slices at the basal, mid-ventricular, and apical levels were acquired using four tissue-characterization sequences: (i) a 5[3]3 MOLLI sequence for native T1 mapping, (ii) a T2-prepared bSSFP sequence for T2 mapping, (iii) a T1ρ-prepared bSSFP sequence for T1ρ mapping, and (iv) the proposed FBmultimap sequence. LGE imaging was obtained 10–15 min after administration of 0.15 mmol/kg gadolinium-based contrast agent. Specific acquisition parameters for each tissue characterization sequence are detailed in Table 1. In the FBmultimap protocol, respiratory motion was corrected using the cross-pair diaphragmatic navigator (dNAV), which dynamically adjusted the imaging plane in real time to avoid the through-plane motion. To counteract the interference of the non-slice-selective preparations on the dNAV signal, an additional slice-selective inversion recovery pulse was applied at the dNAV imaging location and the dNAV was performed before the T2-prep and T1ρ-prep to further reduce the influence of the preparations and maintain stable navigator signal contrast. Residual in-plane motion was further corrected using group-wise image registration [16]. A schematic overview of the complete FBmultimap framework is illustrated in Fig. 1**B**.

**Table 1:** Acquisition parameters for FBmultimap, MOLLI T1, T2-prepared bSSFP, and T1ρ-prepared bSSFP sequences.

Parameters	FBmultimap	MOLLI T1	T2-prepared bSSFP	T1ρ-prepared bSSFP
Echo time (msec)	1.467	1.358	1.347	1.44
Repetition time (msec)	3.08	2.9	2.89	3.02
Flip angle (degree)	45, 35, 70, 50	35	35	30
Field of view (mm^2^)	280 × 320	320 × 360	320 × 360	320 × 360
Acquisition matrix	134 × 192	159 × 256	154 × 192	154 × 192
Spatial resolution (mm²)	2.09 × 1.67	2.01 × 1.41	2.08 × 1.88	2.08 × 1.88
Bandwidth (Hz/pixel)	1200	1200	1000	1200
Slice thickness (mm)	2	2	2	2
Slice gap (%)	150	150	150	150

*FB* free-breathing, *MOLLI* MOdified Look Locker Inversion recovery, *bSSFP* balanced steady state free precession, *T2-prep* T2-prepared, *T1ρ-prep* T1ρ-prepared

### 2.3 Image quality analysis

**Fig. 2:**
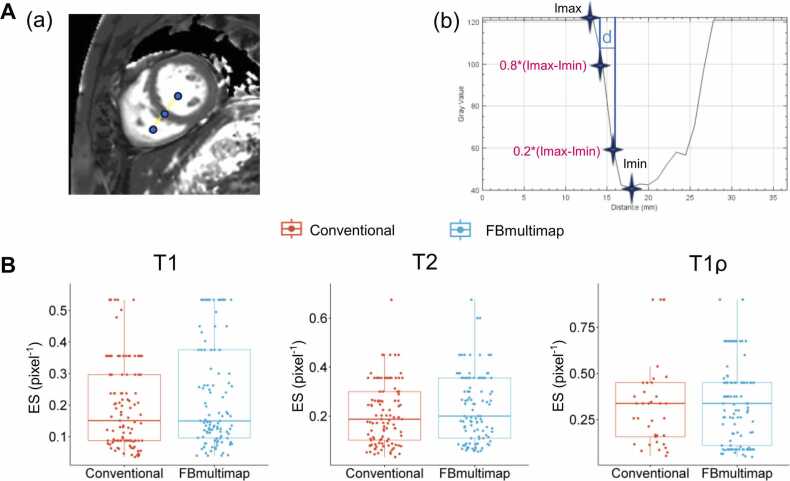
Illustration of ES measurement workflow (**A**) and corresponding ES distributions for T1, T2, and T1ρ mapping sequences (**B**). (**A**) (**a**) Example of a one-dimensional line drawn from the left ventricle to the right ventricle, perpendicular to the septum. (**b**) Example of the SI profile along this line. ES is calculated as the reciprocal of the distance between the points corresponding to 80% and 20% of the intensity range (I_max_ − I_min_). A higher ES value indicates a sharper boundary between the myocardium and the blood pool. **(B)** Boxplots comparing ES between conventional and FBmultimap sequences for T1, T2, and T1ρ maps. Each dot represents an individual measurement. *ES* edge sharpness, *SI* signal intensity, *FBmultimap* free-breathing multimap

Image quality was independently performed using both subjective and objective metrics by two blinded experienced radiologists, X.L.Q. and S.Y.W., with 5 and 10 years of CMR experience, respectively. Subjective image quality was assessed using a four-point Likert scale based on image noise, motion artifacts, distortion, and resolution as follows: 1) poor, non-diagnostic; 2) fair, noticeable motion artifacts or distortion but partially diagnostic; 3) good, mild motion artifacts or distortion; 4) excellent, minimal to no motion artifacts or distortion [9]. Prior to evaluation, both readers underwent a training session to standardize interpretation and ensure consistency in using the Likert scale. In case of scoring disagreement, the final rating was determined by another more experienced reader (Y.Y.C., 15 years of cardiac MRI experience). Edge sharpness (ES) was assessed using ImageJ software (National Institutes of Health, Bethesda, Maryland). Multiple orthogonal line segments were drawn from the LV to the right ventricle across the septum on the mid-ventricular slice to generate signal intensity profiles. ES was defined as the reciprocal of the distance (in pixels⁻¹) between the 20% and 80% intensity points of the signal range (I_max_ – I_min_) along each profile line [18]. The analysis workflow is illustrated in Fig. 2**A**.

### 2.4 Quantitative analysis

All quantitative analyses were conducted using CVI42 software (version 5.17.0, Circle Cardiovascular Imaging, Calgary, Alberta, Canada). Two blinded radiologists (Y.L.W. and X.L.Q.), with 3 and 5 years of experience in CMR, respectively, independently conducted the analyses. LV function parameters, including ejection fraction (EF), end-diastolic volume (EDV), end-systolic volume (ESV), cardiac output (CO), and LV mass, were quantified. For myocardial tissue characterization, both conventional mapping sequences and FBmultimap datasets were analyzed using the dedicated tissue characterization modules (Tissue T1 or T2 mapping) within the software. Endocardial and epicardial contours were delineated according to the Society for Cardiovascular Magnetic Resonance (SCMR) 2020 standardized protocol [19]. To minimize partial volume effects from the blood pool, 10% of the subendocardial and subepicardial borders were manually excluded. Contours were automatically generated on the basal, mid-ventricular, and apical short-axis slices, with manually refined when necessary. Corresponding T1, T2, and T1ρ values were then extracted. For blood pool T1 analysis, circular regions of interest (ROIs) were manually drawn at the center of the LV cavity on basal, mid-ventricular, and apical short-axis slices, carefully excluding papillary muscles and myocardial borders to avoid partial volume effects. The mean T1 value across the three slices was used for subsequent analysis. All quantitative measurements were averaged from both radiologists’ assessments. Inter-observer variability was assessed in all participants (n = 109) for both conventional mapping and FBmultimap sequences by two independent readers. Intra-observer variability was evaluated for both techniques, with Reader 1 independently repeating T1, T2, and T1ρ measurements in 30 randomly selected participants (15 healthy individuals and 15 patients). All measurements were performed in a blinded and temporally separated manner to minimize potential bias.

### 2.5 Statistical analysis

All statistical analyses were performed using SPSS Statistics (version 20.0, IBM Corp., Armonk, New York) and Medcalc (version 23.0, MedCalc Software Ltd., Mariakerke, Belgium). Data normality was assessed using the Shapiro-Wilk test. Normally distributed variables were presented as mean ± SD, and compared using the Student’s *t*-test. Non-normally distributed variables were presented as median and interquartile range (IQR), and compared using the Mann-Whitney *U* test. Categorical variables were presented as counts and percentages, and analyzed using the Chi-square test or Fisher’s exact test, as appropriate. Inter-and intra-observer reproducibility was assessed using the intraclass correlation coefficient (ICC), calculated via a two-way random-effects model with absolute agreement. ICC values were interpreted as follows: <0.50, poor; 0.50–0.75, moderate; 0.75–0.90, good; and ≥0.90, excellent agreement [20]. Agreement between the two imaging techniques was examined with Bland-Altman analysis. All tests were two-tailed, and *p*<0.05 was considered statistically significant.

### 2.6 Diagnostic performance analysis

To evaluate the diagnostic performance of conventional mapping techniques and the FBmultimap in distinguishing LGE-positive (LGE+) and LGE-negative (LGE−) patients from healthy controls, both individual and combined metrics were assessed using receiver operator characteristic (ROC) curve analysis. Combined T1 + T1ρ indices were derived using logistic regression on values from FBmultimap and conventional sequences. ROC curves were then constructed for the following six models: (i) FBmultimap (T1 + T1ρ), (ii) FBmultimap T1, (iii) FBmultimap T1ρ, (iv) conventional (T1 + T1ρ), (v) MOLLI T1, and (vi) T1ρ-prepared bSSFP. The area under the curve (AUC) was calculated to quantify the diagnostic accuracy of each model. Optimal cut-off values were selected by maximizing sensitivity while maintaining specificity ≥70%. Sensitivity, specificity, positive predictive value (PPV), and negative predictive value (NPV) were then calculated.

## 3 Results

### 3.1 Participant characteristics and acquisition time

The study cohort included 33 healthy participants (median age 43, [IQR: 25–57], 19 men), 48 patients with HCM (median age 55, [IQR: 37–61], 32 men), and 28 patients with DCM (median age 52, [IQR: 38–61], 23 men). Baseline characteristics are summarized in Table 2. Compared with the HCM group, the DCM group showed a higher prevalence of heart failure (*p*<0.001) and markedly lower LVEF (24.8 ± 11.2%), compared to both the HCM (60.5 ± 9.5%) and healthy control (59.7 ± 6.1%) cohorts. Both HCM and DCM patients also demonstrated significantly higher LV myocardial mass (162.3 ± 62.5 g and 144.7 ± 45.2 g, respectively) than healthy controls (75.4 ± 16.0 g; *p*<0.001).

**Table 2:** Baseline characteristics of patients and healthy control cohorts.

	HCM patients (*n* = 48)	DCM patients (*n* = 28)	Healthy controls (*n* = 33)	*p*-value
Age (years)^a^	55 [37–61]	52 [38–61]	43 [25–57]	0.058
Gender^b^				0.119
Male	32 (67%)	23 (82%)	19 (58%)	
Female	16 (33%)	5 (18%)	14 (42%)	
BSA (m^2^)^c^	1.8±0.2	1.9±0.3	1.7±0.2	0.001
Medical history^b^				
Coronary artery disease	0 (0%)	0 (0%)	/	/
Hypertension	13 (27%)	11 (39%)	/	0.270
Diabetes mellitus	4 (8%)	4 (14%)	/	0.668
Stoke	1 (2%)	0 (0%)	/	1.000
Heart failure	3 (6%)	14 (50%)	/	< 0.001
Ventricular arrhythmia	13 (27%)	10 (36%)	/	0.429
Syncope	4 (8%)	0 (0%)	/	0.300
NYHA heart failure classification^b^				
I	22 (46%)	10 (36%)	/	0.389
II	19 (40%)	13 (46%)	/	0.560
III	7 (14%)	4 (14%)	/	1.000
IV	0 (0%)	1 (4%)	/	0.368
Heart rate^c^	68.2±9.8	79.2±16.0	74.0±10.8	< 0.001
LV function parameters^c^				
EF (%)	60.5±9.5	24.8±11.2	59.7±6.1	< 0.001
EDV (mL)	144.5±27.9	246.8±69.5	124.0±23.7	< 0.001
ESV (mL)	57.2±17.4	186.3±59.5	50.5±13.8	< 0.001
CO (L/min)	5.9±1.4	4.7±2.2	5.4±0.8	0.008
LV myocardial mass (g)^c^	162.3±62.5	144.7±45.2	75.4±16.0	< 0.001
LGE presence^b^	34 (71%)	17 (61%)	/	0.365

*HCM* hypertrophic cardiomyopathy, *DCM* dilated cardiomyopathy, *BSA* body surface area, *NYHA* New York Heart Association, *LV* left ventricular, *EF* ejection fraction, *EDV* end-diastolic volume, *ESV* end-systolic volume, *CO* cardiac output, *LGE* late gadolinium enhancement

a Data are medians with interquartile ranges in parentheses

b Data are the number of participants with the percentage in parentheses

c Data are means ± standard deviations

Scan time analysis showed that individual breath-hold acquisitions, including MOLLI T1 (56 ± 4 s, *n* = 109), T2-prepared (46 ± 2 s, *n* = 109) and T1ρ-prepared (62 ± 6 s, *n* = 37) bSSFP, were slightly shorter than the FBmultimap sequence (66 ± 6 s, *n* = 109; *p*<0.001). However, FBmultimap significantly reduced overall scan time compared to combined conventional mapping sequences: 117 ± 6 s for T1 + T2 mapping (*n* = 109; *p*<0.001), and 195 ± 10 s for T1 + T2 + T1ρ mapping (*n* = 37; *p*<0.001).

### 3.2 Image quality comparison of FBmultimap and conventional mapping

Table 3 summarizes both subjective and objective image quality metrics for FBmultimap and conventional sequences.

**Table 3:** Comparison of subjective and objective image quality between FBmultimap and conventional mapping techniques.

Sequences	Techniques	Four-point Likert scale (%)				ES (pixels⁻¹)	*p* (Likert)	*p* (ES)
		Score 1	Score 2	Score 3	Score 4			
T1	FBmultimap	1	8	45	46	0.15 (0.09 ∼ 0.30)	0.066	0.637
	Conventional	2	7	29	62	0.15 (0.10 ∼ 0.38)		
T2	FBmultimap	0	5	37	58	0.20 (0.10 ∼ 0.31)	0.613	0.065
	Conventional	1	5	42	52	0.20 (0.11 ∼ 0.36)		
T1ρ	FBmultimap	0	6	33	61	0.34 (0.14 ∼ 0.45)	0.090	0.614
	Conventional	5	3	35	57	0.34 (0.11 ∼ 0.45)		

Subjective image quality is shown as score percentages (1 = non-diagnostic, 4 = excellent), and ES as median (IQR) measured at the mid-ventricular slice with higher values indicating sharper boundaries.

*FB* free-breathing, *ES* edge sharpness, *IQR* interquartile range

**Fig. 3:**
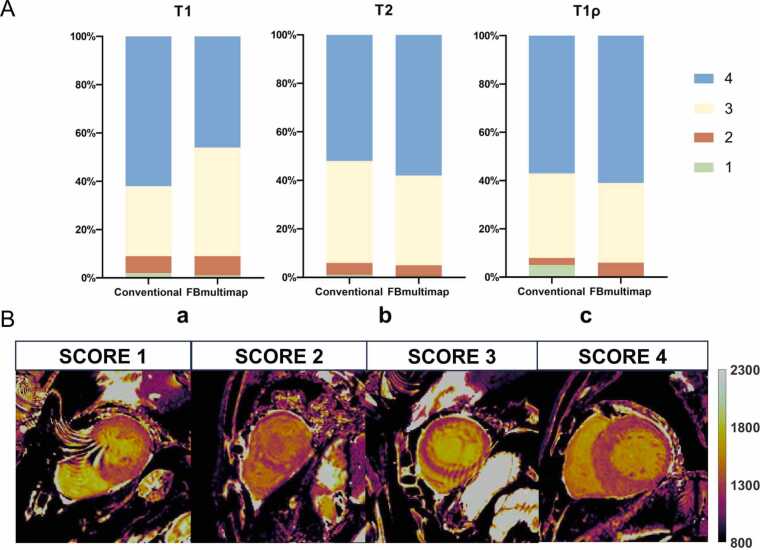
Distribution of image quality scores for mapping sequences (**A**) and representative examples of scoring criteria from 1 to 4 (**B**). (**A**) Distribution of image quality scores using a four-point Likert scale for FBmultimap and conventional maps sequences. (**a**) MOLLI T1 compared with T1 obtained from FBmultimap. (**b**) T2-prepared bSSFP compared with T2 obtained from FBmultimap. (**c**) T1ρ-prepared bSSFP compared with T1ρ obtained from FBmultimap. (**B**) Representative examples of image quality grading from score 1 to score 4. Score 1 represents non-diagnostic image quality, score 2 indicates fair quality, score 3 reflects good quality, and score 4 denotes excellent quality. *FBmultimap* free-breathing multimap, *MOLLI* MOdified Look Locker Inversion recovery, *bSSFP* balanced steady state free precession

In the subjective assessment, inter-reader discrepancies were observed in 23 cases and resolved by a third, blinded reader (Table S1). As shown in Fig. 3**A**, there were no statistically significant differences in the Likert score distributions between FBmultimap and conventional sequences, including MOLLI T1 (*p *= 0.066), T2-prepared bSSFP (*p *= 0.613), and T1ρ-prepared bSSFP (*p *= 0.090). Across all sequences and techniques, more than 95% of scans were rated as diagnostic (Likert score ≥ 2), as detailed in Table 3. Representative scoring examples (Likert 1–4) are shown in Fig. 3**B**.

For the objective assessment, ES measured at the mid-ventricular slice did not differ significantly between the two sequences for T1, T2, or T1ρ (all *p*>0.05). Median ES values (pixel^-1^) were comparable: T1 (0.15 [IQR: 0.09–0.30] vs. 0.15 [IQR: 0.10–0.38], *p *= 0.637), T2 (0.20 [IQR:0.10–0.31] vs. 0.20 [IQR: 0.11–0.36], *p *= 0.065), and T1ρ (0.34 [IQR: 0.14–0.45] vs. 0.34 [IQR: 0.11–0.45], *p *= 0.614). Full distribution patterns are shown in Fig. 2**B**.

**Fig. 4:**
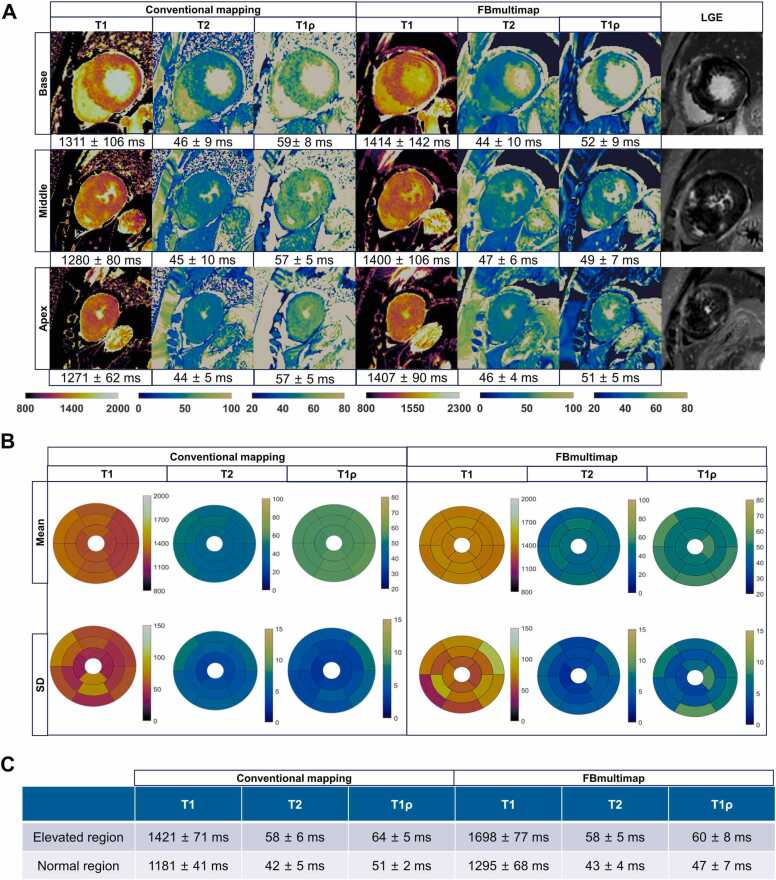
Representative images from conventional mapping, FBmultimap, and LGE in a 61-year-old female patient with HCM. (**A**) The LGE image demonstrates multiple patchy areas of enhancement involving the basal, mid, and apical segments of left ventricular wall. Corresponding conventional mapping and FBmultimap show markedly elevated T1 and T1ρ values in the affected myocardial regions. The image quality score for FBmultimap T2 was 3 due to slight signal loss in the interventricular septum on the basal slice, while all other maps were graded as 4. (**B**) AHA 16-segment bullseye plots showing myocardial T1, T2, and T1ρ values for conventional mapping and FBmultimap techniques. Top rows show mean values, and bottom rows show SD. (**C**) Quantitative relaxation times of the elevated and normal regions were measured on the mid-ventricular slice and are summarized in the table. *FBmultimap* free-breathing multimap, *LGE* late gadolinium enhancement, *HCM* hypertrophic cardiomyopathy, *SD* standard deviations

Representative images from both conventional mapping and FBmultimap in patients with HCM are presented in Fig. 4**A**, along with corresponding AHA 16-segment bullseye plots (Fig. 4**B**) for regional comparison. In this example, both techniques showed higher T1 and T1ρ values in LGE+ regions compared to remote myocardium on the mid-ventricular slice (Fig. 4**C**).

### 3.3 Quantitative comparison of FBmultimap and conventional mapping

As shown in Table 4 and Table S2, all mapping techniques demonstrated good to excellent inter- and intra-observer agreement, with ICC values exceeding 0.80 in both patient and healthy cohorts.

**Table 4:** Inter-observer agreement of myocardial T1, T2, and T1ρ measurements using conventional mapping and FBmultimap techniques in patient and healthy cohorts.

	Sequence	Reader 1 (ms)^a^	Reader 2 (ms)^a^	ICC
Patient	MOLLI T1	1220.4±54.3	1221.0±54.1	0.999
	T2-prepared bSSFP	43.1±3.9	43.2±4.0	0.969
	T1ρ-prepared bSSFP	55.6±3.8	55.4±3.9	0.969
	FBmultimap T1	1292.3±73.6	1293.0±73.6	0.999
	FBmultimap T2	42.9±3.5	43.0±3.6	0.989
	FBmultimap T1ρ	49.6±4.3	50.0±4.3	0.962
Healthy	MOLLI T1	1176.8±42.3	1176.5±45.5	0.967
	T2-prepared bSSFP	42.5±3.2	42.2±2.5	0.804
	T1ρ-prepared bSSFP	52.4±2.7	52.6±2.5	0.981
	FBmultimap T1	1217.0±45.4	1218.2±47.2	0.945
	FBmultimap T2	41.9±2.9	41.9±2.6	0.952
	FBmultimap T1ρ	44.1±3.7	45.0±3.3	0.972

*FB* free-breathing, *ICC* intraclass correlation coefficient, *MOLLI* MOdified Look Locker Inversion recovery, *bSSFP* balanced steady state free precession

a Data are means ± standard deviations

In the patient cohort, there was no significant difference in T2 values between FBmultimap and T2-prepared bSSFP (42.9 ± 3.5 ms vs. 43.1 ± 3.9 ms; *p *= 0.741). In contrast, FBmultimap yielded significantly higher T1 values compared to MOLLI (1292.7 ± 73.6 ms vs. 1220.7 ± 54.2 ms; *p*<0.001), and significantly lower T1ρ values compared to T1ρ-prepared bSSFP (49.8 ± 4.2 ms vs. 55.5 ± 3.8 ms; *p*<0.001). Similar trends were observed in the healthy cohort: no significant difference in T2 values (41.9 ± 2.7 ms vs. 42.4 ± 2.7 ms; *p *= 0.574), but significantly higher T1 values (1217.6 ± 45.6 ms vs. 1176.7 ± 43.6 ms; *p*<0.001) and lower T1ρ values (44.5 ± 3.5 ms vs. 52.5 ± 2.6 ms; *p *= 0.002) with FBmultimap compared to conventional mapping techniques. The mean LV blood pool T1 was significantly lower with FBmultimap compared to MOLLI (1733.0 ± 93.8 ms vs. 1802.5 ± 95.4 ms, *p*<0.001). The reduction was consistent across base, middle, and apex levels, with detailed values provided in Table S3.

**Fig. 5:**
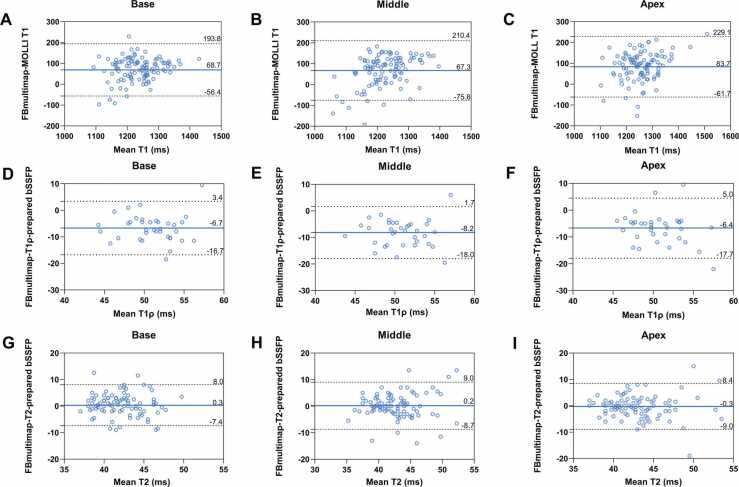
Bland-Altman analysis comparing FBmultimap with conventional mapping sequences in basal, mid, and apical slices. (**A-C**) compare T1 values between FBmultimap and the MOdified Look Locker Inversion recovery (MOLLI) sequence. **(D-F**) compare T1ρ values between FBmultimap and T1ρ-prepared bSSFP. (**G-I**) compare T2 values between FBmultimap and T2-prepared bSSFP. The mean difference is indicated by the solid blue line. The 95% limits of agreement are indicated by the two dotted black lines. *FBmultimap* free-breathing multimap, *MOLLI* MOdified Look Locker Inversion recovery, *bSSFP* balanced steady state free precession

Bland-Altman analysis (Fig. 5) across all participants revealed systematic biases in T1 measurements between FBmultimap and MOLLI: 68.7, 67.3, and 83.7 ms in basal, mid, and apical segments, respectively. Corresponding biases in T1ρ were −6.7, −8.2, and −6.4 ms. T2 values showed minimal biases, with differences of 0.3, 0.2, and −0.3 ms, respectively.

### 3.4 Applications of FBmultimap and conventional mapping across cohorts

The normal ranges for T1, T2, and T1ρ values of conventional mapping and FBmultimap derived from the healthy volunteer cohort are shown in Table S4.

**Fig. 6:**
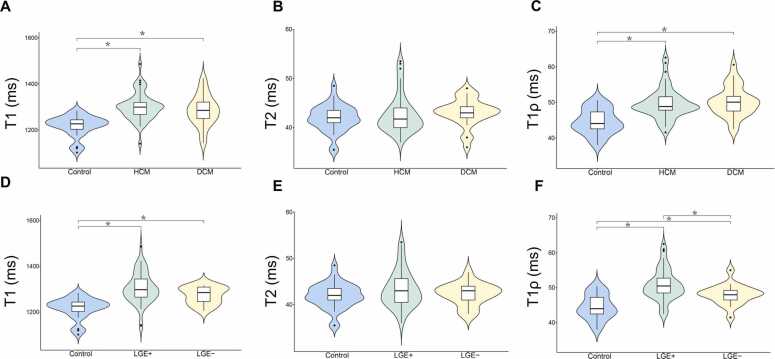
Violin plots showing myocardial T1, T2, and T1ρ values measured using the FBmultimap. (**A-C**) Comparisons between healthy controls and patients with HCM or DCM, including (**A**) T1, (**B**) T2, and (**C**) T1ρ values. (**D-F**) Subgroup analysis based on the presence or absence of LGE, showing (**D**) T1, (**E**) T2, (**F**) T1ρ values across control, LGE+, and LGE− groups. Statistically significant differences are indicated by asterisks (*, *p*<0.05). *FBmultimap* free-breathing multimap, *HCM* hypertrophic cardiomyopathy, *DCM* dilated cardiomyopathy, *LGE* late gadolinium enhancement, *LGE+* LGE-positive, *LGE−* LGE-negative

Fig. 6 **and** Table 5 show the distribution of T1, T2, and T1ρ values measured using the FBmultimap technique across the three cohorts. Both T1 and T1ρ values were significantly elevated in the HCM and DCM groups compared to healthy controls (all *p*<0.001). In contrast, T2 values did not show significant differences among HCM, DCM, and control groups.

**Table 5:** Tissue characterization using FBmultimap across different subgroups.

	FBmultimap T1 (ms)^a^	FBmultimap T2 (ms)^a^	FBmultimap T1ρ (ms)^a^
*Mapping values*			
Control	1217.6±45.6	41.9±2.7	44.5±3.5
HCM	1301.7±60.6	42.8±4.0	49.6±4.3
DCM	1277.3±91.0	43.1±2.8	50.2±4.2
LGE+	1298.4±82.6	43.4±4.0	50.8±4.5
LGE−	1273.3±37.3	42.3±2.4	48.0±2.8
*p-values*			
HCM vs. Control	<0.001	0.872	<0.001
DCM vs. Control	<0.001	0.054	<0.001
LGE+ vs. Control	<0.001	0.202	<0.001
LGE− vs. Control	<0.001	0.572	0.001
LGE+ vs. LGE−	0.054	0.611	0.006

*FBmultimap* free-breathing multimap, *HCM* hypertrophic cardiomyopathy, *DCM* dilated cardiomyopathy, *LGE* late gadolinium enhancement

a Data are means ± standard deviations

Further subgroup analysis based on the presence or absence of the LGE revealed that both LGE+ and LGE− groups exhibited significantly higher T1 and T1ρ values than healthy controls (all *p*<0.05). However, no significant differences were observed in T2 values among the LGE+, LGE−, and control groups.

Similar patterns were observed using conventional mapping techniques, including MOLLI T1, T2-prepared bSSFP, and T1ρ-prepared bSSFP, as illustrated in Fig. S1 **and** Table S5.

### 3.5 Comparison of diagnostic performance between FBmultimap and conventional mapping

**Fig. 7:**
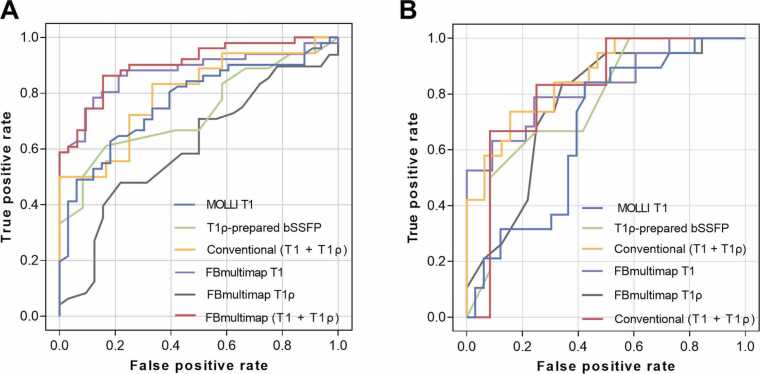
ROC curves comparing the diagnostic performance of FBmultimap and conventional mapping techniques. The ROC curves demonstrate the diagnostic performance of FBmultimap (T1 + T1ρ), conventional (T1 + T1ρ), FBmultimap T1, FBmultimap T1ρ, conventional MOLLI T1, and T1ρ-prepared bSSFP. Diagnostic discrimination is shown for differentiating patients with late gadolinium enhancement (LGE+, **A**) and without late gadolinium enhancement (LGE−, **B**) from healthy controls. *ROC* receiver operator characteristic, *FBmultimap* free-breathing multimap, *MOLLI* MOdified Look Locker Inversion recovery, *bSSFP* balanced steady state free precession

The diagnostic performance of FBmultimap and conventional mapping techniques is summarized in Table 6 and Fig. 7. In differentiating both LGE+ and LGE− patients from healthy controls, FBmultimap T1 achieved higher AUCs than MOLLI T1 (0.877 vs. 0.770 for LGE+, and 0.829 vs. 0.671 for LGE−), whereas FBmultimap T1ρ yielded lower AUCs than T1ρ-prepared bSSFP (0.608 vs. 0.734 for LGE+, and 0.764 vs. 0.778 for LGE−). Among all models, the combined FBmultimap index (T1 + T1ρ) achieved the highest diagnostic accuracy, with AUCs of 0.904 for LGE+ and 0.859 for LGE−. These values were higher than those obtained with FBmultimap T1, FBmultimap T1ρ, MOLLI T1, T1ρ-prepared bSSFP, and the conventional combined index (T1 + T1ρ) (0.801 and 0.819). Table S6 provides a supplementary comparison between (T1 + T1ρ) and (T1 + T2) indices derived from FBmultimap.

**Table 6:** Diagnostic performance of FBmultimap and conventional mapping techniques for distinguishing LGE+ and LGE− patients from healthy controls.

	Sequences	AUC	Optimal cut-off	Sensitivity (%)	Specificity (%)	PPV (%)	NPV (%)
LGE+	FBmultimap (T1 + T1ρ)	0.904	0.66	86.3	84.4	89.8	79.4
	Conventional (T1 + T1ρ)	0.801	0.49	83.3	66.7	78.9	72.7
	FBmultimap T1ρ	0.608	42.00	48.0	78.1	76.7	50.0
	FBmultimap T1	0.877	1257.00	78.4	87.9	90.9	72.5
	T1ρ-prepared bSSFP	0.734	54.00	61.1	83.3	84.6	58.8
	MOLLI T1	0.770	1217.00	62.8	81.8	84.2	58.7
LGE−	FBmultimap (T1 + T1ρ)	0.859	0.49	73.7	84.4	73.7	84.4
	Conventional (T1 + T1ρ)	0.819	0.33	83.3	75.0	62.5	90.0
	FBmultimap T1ρ	0.764	45.50	84.2	65.6	59.3	87.5
	FBmultimap T1	0.829	1244.00	79.0	75.8	65.2	86.2
	T1ρ-prepared bSSFP	0.778	54.00	66.7	83.3	66.7	83.3
	MOLLI T1	0.671	1172.50	84.2	57.6	53.3	86.4

*LGE* late gadolinium enhancement, *AUC* area under the curve, *PPV* positive predictive value, *NPV* negative predictive value, *FB* free-breathing, *bSSFP* balanced steady state free precession, *MOLLI* MOdified Look Locker Inversion recovery

The combined FBmultimap index also demonstrated the most balanced diagnostic metrics: for the LGE+ group, sensitivity, specificity, PPV, and NPV were 86.3%, 84.4%, 89.8%, and 79.4%, respectively. For the LGE− group, the corresponding values were 73.7%, 84.4%, 73.7%, and 84.4%. These results were generally favorable compared to those obtained with other tested models.

## 4 Discussion

In this prospective study, we evaluated the feasibility and diagnostic performance of FBmultimap for the simultaneous acquisition of co-registered myocardial T1, T2, and T1ρ maps in 48 patients with HCM, 28 with DCM, and 33 healthy controls. FBmultimap successfully provided simultaneous mapping of all three parameters with image quality comparable to conventional techniques, significantly reduced scan time, high inter-observer reproducibility, and consistent diagnostic capability. Although systematic differences in absolute T1 and T1ρ values were observed between FBmultimap and conventional methods, the diagnostic trends across disease cohorts were preserved. Both FBmultimap and conventional mapping demonstrated significantly elevated T1 and T1ρ values in HCM and DCM patients compared to healthy controls, while T2 values remained unchanged. Notably, the combined FBmultimap index (T1 + T1ρ) achieved the highest diagnostic accuracy for distinguishing both LGE+ and LGE− patients from healthy individuals, supporting its robustness and potential clinical applicability across diverse myocardial diseases.

FBmultimap introduces several technical innovations that address key limitations of conventional cardiac mapping techniques [16]. First, it enables fully free-breathing data acquisition in significantly shorter scan time, eliminating the need for multiple breath-holds, an important advantage for cardiomyopathy patients who have difficulty sustaining repeated breath-holds due to heart failure symptoms [21]. Second, FBmultimap acquires all parametric images (T1, T2, T1ρ) within a single session, ensuring spatial and temporal co-registration. This integration facilitates the complementary assessment of myocardial microstructure information and enhances diagnostic precision [16], [22]. Third, unlike heart rate-dependent approaches such as MOLLI and T2-/T1ρ-prepared bSSFP, FBmultimap utilizes a dictionary-based framework that eliminates idle cardiac cycles and accounts for cross-parameter interactions within the signal model. This strategy improves quantitative accuracy and robustness, particularly in patients with arrhythmias or variable heart rates [23], [24]. Collectively, these features make FBmultimap a more efficient, robust, and clinically accessible tool for comprehensive myocardial tissue characterization.

Although previous studies have demonstrated the feasibility of advanced multiparametric mapping techniques, such as joint T1/T2 mapping and cMRF, most investigations were conducted using phantom models. The first large-scale in vivo study was conducted by Hamilton *et al.*, involving 58 healthy volunteers. While the study demonstrated reliable image quality with cMRF, the absence of patient data limited its clinical applicability [25]. A few studies have extended these methods to small patient cohorts. For example, Eck *et al.* evaluated cMRF in nine patients with cardiac amyloidosis and reported high diagnostic accuracy [26]. Similarly, Cavallo *et al.* demonstrated that cMRF identified elevated T1 values in nine patients with NICM compared to healthy controls using a 1.5T scanner [27]. However, these studies were constrained by their small sample sizes. A conference report applied cMRF to a more diverse cohort of 175 participants, including patients with ischemic and non-ischemic cardiomyopathy as well as healthy controls. The study demonstrated good agreement between cMRF and conventional mapping, suggesting feasibility in more heterogeneous populations [28]. In contrast to these prior research, our study focused on a disease-specific cohort and employed a technically distinct approach. We provide robust clinical evidence supporting the feasibility of FBmultimap in a large and diverse cohort, comprising 48 patients with HCM, 28 with DCM, and 33 healthy controls. Moreover, FBmultimap incorporates correction for B1 field inhomogeneities directly into its dictionary-based signal model, thereby improving the accuracy of parameter estimation. This approach mitigates the quantitative biases frequently encountered in joint T1/T2 mapping and cMRF due to radiofrequency non-uniformities [16].

Consistent with previous findings by Lyu *et al.* [16], we observed systematic differences in absolute T1 and T1ρ values between FBmultimap and conventional methods. Specifically, FBmultimap yielded higher T1 values compared to MOLLI, and lower T1ρ values compared to T1ρ-prepared bSSFP. These trends align with prior observations in healthy subjects (T1: 1218 ± 50 ms vs. 1166 ± 38 ms; T1ρ: 45.3 ± 4.4 ms vs. 50.2 ± 4.0 ms). These discrepancies may be attributed to intrinsic technical differences between the mapping methods. MOLLI is known to underestimate T1 values, especially in settings of prolonged T1 relaxation (>1000 ms) and elevated heart rates (>80 bpm), due to factors such as suboptimal inversion efficiency, magnetization transfer effects, and heart rate dependence. In contrast, FBmultimap derives parametric maps through dictionary matching, with comprehensive simulation of the entire acquisition process during dictionary generation. This design renders the method independent of heart rate, thereby improving quantitative accuracy, and eliminates the need for inserting dummy cardiac cycles to allow sufficient T1 relaxation, thus enhancing acquisition efficiency [29]. Additionally, we observed a greater T1 bias in apical segments compared to basal and mid-ventricular regions, likely due to partial volume effects and susceptibility artifacts from left ventricular curvature and trabeculation [30]. T1ρ values measured by FBmultimap were lower than those from conventional two-parameter fitting-based T1ρ-prepared bSSFP technique, which may be explained by the overestimation of T1ρ in the latter method due to incomplete T1 recovery during the acquisition process [31]. These observations suggest that the absolute value differences between FBmultimap and conventional techniques stem from systematic technical variation, rather than biological inconsistency. Notably, FBmultimap yielded lower blood T1 values compared to MOLLI. This discrepancy is likely attributable to magnetization effects from the cross-pair diaphragmatic navigator, which may perturb inflowing venous blood signal. While this did not affect myocardial T1 quantification, it could impact the accuracy of derived extracellular volume (ECV) estimates. To address this, a new FBmultimap sequence incorporating the pencil-beam navigator is currently under development.

To determine whether the observed differences in T1 and T1ρ values influence diagnostic accuracy, FBmultimap was evaluated in clinical cohorts of patients with HCM and DCM. The parametric values obtained from FBmultimap followed trends consistent with previous single-parameter studies, showing elevated T1 and T1ρ values in fibrotic HCM and DCM regardless of LGE status, while T2 values remained unchanged. This supports the feasibility of FBmultimap in vivo multiparametric myocardial tissue characterization across different disease states. While previous studies have assessed T1 and T1ρ mapping separately [3], [32], this study is the first to assess their combined diagnostic potential using a co-registered, free-breathing framework. The composite FBmultimap index (T1 + T1ρ) yielded the highest diagnostic performance, with AUCs of 0.904, and 0.859 for distinguishing LGE+ and LGE− patients from controls, respectively. Specifically, FBmultimap T1 alone outperformed MOLLI T1, suggesting that improved T1 quantification substantially contributed to the overall diagnostic accuracy, and composite FBmultimap index incorporating T1ρ further enhanced performance beyond T1 alone. While T1 reflects broader interstitial expansion, T1ρ is particularly sensitive to collagen-rich fibrosis. The combination of these complementary parameters allowed more comprehensive detection of early or diffuse myocardial abnormalities that may be overlooked when relying on a single metric [33]. While Pang et al. recently modeled adiabatic T1ρ as a weighted combination of T1 and T2 [34], it remains unclear whether T1ρ provides independent diagnostic value beyond these conventional parameters. To explore this, we performed a supplementary analysis comparing the diagnostic performance of two composite indices: (T1 + T1ρ) versus (T1 + T2). The (T1 + T1ρ) index demonstrated superior AUCs in both LGE+ (0.904 vs. 0.860) and LGE− (0.859 vs. 0.843) groups. These results suggest that T1ρ offers stronger and more complementary contrast relative to T2, especially considering the limited group-level differences observed in T2. Thus, despite its theoretical mathematical dependence, T1ρ appears to retain independent clinical value. Nonetheless, as T1ρ quantification remains sensitive to sequence design and acquisition parameters, further standardization is needed to ensure reproducibility. FBmultimap (T1 + T1ρ) also demonstrated balanced diagnostic metrics, with sensitivity, specificity, PPV, and NPV all exceeding 70%. Notably, it achieved high sensitivity (73.7%) and specificity (84.4%) in distinguishing LGE− patients from controls, highlighting its potential to detect diffuse interstitial fibrosis or early fibrotic changes that may be undetectable on LGE imaging [3]. These findings indicate that FBmultimap not only retains the diagnostic utility of established techniques but may also provide more physiologically accurate T1 and T1ρ quantification. Its fully free-breathing acquisition, co-registered multiparametric maps, and robust diagnostic performance across LGE subgroups support its use as a clinically feasible tool for early fibrosis detection, risk stratification, and disease monitoring in NICM populations [35].

## 5 Limitations

Several limitations should be noted. First, although the study included well-characterized patient cohorts and healthy controls, it was conducted at a single center. Larger, multicenter studies are warranted to confirm generalizability. Second, genetic testing was not performed, which may limit the ability to definitively distinguish HCM from phenocopies such as hypertensive cardiomyopathy. Third, T1ρ-prepared bSSFP mapping was not performed in the DCM group, and ECV was not assessed. Fourth, patients with severe arrhythmia or complex cardiac conditions were excluded, limiting assessment of FBmultimap under challenging acquisition scenarios. Fifth, to reduce computational demand, dictionary generation used simplified Bloch simulations without accounting for slice profile or inversion pulse imperfections, which may cause minor biases in parameter estimation. Moreover, real-time slice tracking relied on fixed foot-head correction only, potentially limiting accuracy under complex respiratory motion. Finally, our study focused on HCM and DCM; future work should evaluate FBmultimap in broader disease contexts, such as myocarditis, cardiac amyloidosis, and iron overload, to validate its capability in quantifying edema, fibrosis, and other myocardial abnormalities.

## 6 Conclusion

In conclusion, we demonstrated that FBmultimap enables rapid, free-breathing acquisition of co-registered T1, T2, and T1ρ maps in patients with HCM and DCM, outperforming single-parameter conventional MOLLI T1 and T1ρ-prepared bSSFP in the detection of myocardial fibrosis while reducing scan time. Although a systematic T1 and T1ρ bias was observed, it did not affect the technique’s ability to detect relative pathological changes, underscoring its diagnostic robustness and potential clinical utility.

## Funding

Joint Research Development Project between Shenkang and United Imaging on Clinical Research and Translation (SKLY2022CRT201). The Shanghai Municipal “Explorer Plan” (the second batch) project in 2024 (24TS1411000). Fujian Provincial Natural Science Foundation of China (2022J05333). Shanghai Pujiang Program (21PJD012). The Explorer Program of Shanghai Municipality (23TS1400300).

## Author contributions

**Yali Wu:** Writing – original draft, Validation, Data curation. **Xianling Qian:** Writing – review & editing, Methodology, Data curation, Conceptualization. **Kai Liu:** Visualization, Methodology, Data curation. **Zhenfeng Lyu:** Visualization, Validation, Software. **Shiyu Wang:** Validation, Investigation, Conceptualization. **Yinyin Chen:** Supervision, Project administration, Methodology. **Ling Chen:** Visualization, Data curation. **Zhuolin Liu:** Visualization, Investigation, Data curation. **Lin Tian:** Visualization, Software. **Hang Jin:** Supervision, Resources, Methodology. **Haikun Qi:** Visualization, Software, Resources. **Mengsu Zeng:** Writing – review & editing, Supervision, Resources, Methodology, Conceptualization.

## Declaration of competing interests

The authors declare that they have no known competing financial interests or personal relationships that could have appeared to influence the work reported in this paper.

## Data sharing statement

Data generated or analyzed during the study are available from the corresponding author upon request.
