# Supplementary material for: Simultaneous free-breathing T1, T2, and T1ρ mapping for myocardial fibrosis detection in non-ischemic cardiomyopathy: A comparative study with conventional techniques
Source: J Cardiovasc Magn Reson. 2025 Nov 3;27(2):101982. doi: 10.1016/j.jocmr.2025.101982 (PMC12766603; doi:10.1016/j.jocmr.2025.101982)
Supplement: Supplementary file 1 — Supplementary material [file mmc1.docx]

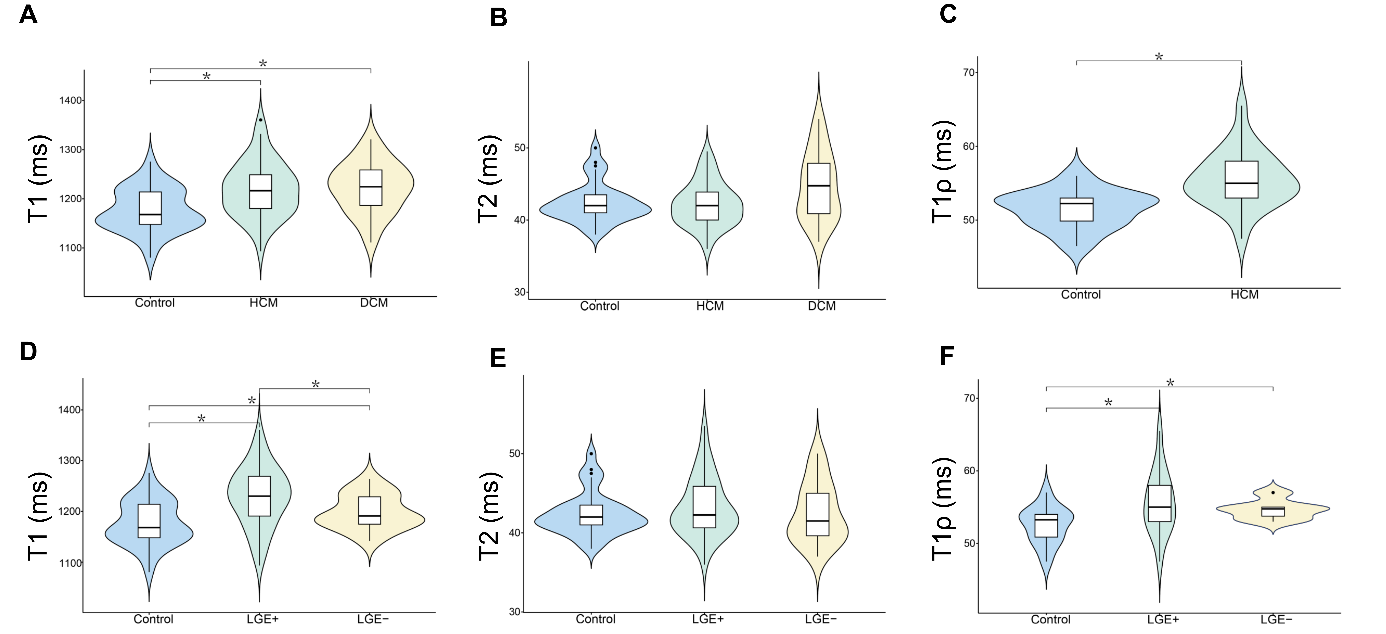


**Figure S1.** Violin plots showing myocardial relaxation times using conventional mapping sequences, including MOdified Look Locker Inversion recovery (MOLLI) T1, T2-prepared balanced steady-state free precession (bSSFP), and T1ρ-prepared bSSFP. **(A-C)** Comparisons between healthy controls and patients with hypertrophic cardiomyopathy (HCM) or dilated cardiomyopathy (DCM), including **(A)** T1, **(B)** T2, and **(C)** T1ρ values. **(D-F)** Subgroup analysis based on the presence or absence of late gadolinium enhancement (LGE), showing **(D)** T1, **(E)** T2, and **(F)** T1ρ values across control, LGE-positive (LGE+), and LGE-negative (LGE−) groups. Statistically significant differences are indicated by asterisks (*, *p* < 0.05).

**Table S1:** Summary of inter-reader disagreements and final adjudicated scores

| Case ID | Technique | Sequence | Reader 1 score | Reader 2 score | Δ Score | Reason for disagreement | Final score |
| --- | --- | --- | --- | --- | --- | --- | --- |
| 7 | Conventional | T1 | 3 | 4 | 1 | Motion artifact | 4 |
| 11 | FBmultimap | T1 | 4 | 3 | 1 | Motion artifact | 3 |
| 17 | Conventional | T2 | 2 | 3 | 1 | mixed artifact | 2 |
| 18 | FBmultimap | T1 | 4 | 3 | 1 | Distortion | 3 |
| 31 | FBmultimap | T1ρ | 3 | 4 | 1 | Motion artifact | 3 |
| 38 | Conventional | T1 | 3 | 2 | 1 | Distortion | 3 |
| 39 | FBmultimap | T1ρ | 3 | 2 | 1 | Distortion | 3 |
| 46 | FBmultimap | T2 | 5 | 4 | 1 | Motion artifact | 4 |
| 46 | FBmultimap | T1ρ | 4 | 3 | 1 | Distortion | 3 |
| 52 | Conventional | T1 | 4 | 3 | 1 | Motion artifact | 4 |
| 54 | FBmultimap | T1 | 3 | 4 | 1 | Distortion | 4 |
| 62 | Conventional | T2 | 4 | 3 | 1 | Motion artifact | 4 |
| 75 | FBmultimap | T1ρ | 4 | 3 | 1 | Motion artifact | 4 |
| 75 | Conventional | T1ρ | 4 | 3 | 1 | Motion artifact | 4 |
| 77 | Conventional | T2 | 3 | 2 | 1 | mixed artifact | 3 |
| 81 | FBmultimap | T2 | 3 | 4 | 1 | Motion artifact | 4 |
| 86 | Conventional | T1 | 2 | 3 | 1 | mixed artifact | 2 |
| 86 | FBmultimap | T1ρ | 3 | 4 | 1 | Motion artifact | 4 |
| 91 | Conventional | T2 | 3 | 4 | 1 | Motion artifact | 3 |
| 99 | FBmultimap | T1ρ | 3 | 2 | 1 | Distortion | 3 |
| 101 | Conventional | T1ρ | 4 | 3 | 1 | Motion artifact | 4 |
| 104 | FBmultimap | T2 | 4 | 3 | 1 | Motion artifact | 4 |
| 105 | Conventional | T1ρ | 3 | 4 | 1 | Motion artifact | 4 |

Δ Score represents the absolute difference between the two readers' scores. Final scores were determined by an independent third reader. Reasons for disagreement were categorized as motion artifact, distortion, or mixed artifact (a combination of motion artifact and distortion).

Note: FB = free-breathing.

**Table S2:** Intra-observer agreement of myocardial T1, T2, and T1ρ measurements using FBmultimap techniques in patient and healthy cohorts

|  | Sequence | Measurement 1 (ms) | Measurement 2 (ms) | ICC |
| --- | --- | --- | --- | --- |
| Patient | MOLLI T1 | 1203.5 ± 37.2 | 1204.0 ± 35.7 | 0.997 |
|  | T2-prepared bSSFP | 40.4 ± 2.3 | 40.0 ± 2.6 | 0.958 |
|  | T1ρ-prepared bSSFP | 54.8 ± 3.4 | 54.8 ± 3.6 | 0.941 |
|  | FBmultimap T1 | 1300.1 ± 39.5 | 1300.5 ± 37.8 | 0.997 |
|  | FBmultimap T2 | 40.4 ± 2.2 | 40.7 ± 2.6 | 0.860 |
|  | FBmultimap T1ρ | 46.7 ± 2.6 | 45.9 ± 2.3 | 0.903 |
| Healthy | MOLLI T1 | 1165.8 ± 45.3 | 1165.7 ± 46.3 | 0.997 |
|  | T2-prepared bSSFP | 42.1 ± 3.9 | 42.5 ± 4.1 | 0.984 |
|  | T1ρ-prepared bSSFP | 52.6 ± 3.0 | 52.5 ± 3.3 | 0.979 |
|  | FBmultimap T1 | 1217.5 ± 55.9 | 1216.8 ± 54.1 | 0.997 |
|  | FBmultimap T2 | 41.7 ± 2.0 | 42.3 ± 1.8 | 0.883 |
|  | FBmultimap T1ρ | 43.6 ± 4.1 | 43.8 ± 4.0 | 0.947 |

Measurements were performed by the same reader (Reader 1) on a randomly selected subset of 30 participants (15 healthy subjects and 15 patients) to assess intra-observer agreement.

Note: FB = free-breathing, ICC = intraclass correlation coefficient, MOLLI = MOdified Look Locker Inversion recovery, bSSFP = balanced steady state free precession.

**Table S3.** Native LV blood pool T1 values measured by FBmultimap and MOLLI at base, middle, and apex levels

|  | FBmultimap T1 (ms) | MOLLI T1 (ms) | *p-values* |
| --- | --- | --- | --- |
| Base | 1725.5 ± 94.2 | 1793.6 ± 98.2 | < 0.001 |
| Middle | 1730.9 ± 107.7 | 1807.4 ± 95.9 | < 0.001 |
| Apex | 1745.9 ± 99.4 | 1811.6 ± 103.8 | < 0.001 |

Note: FB = free-breathing, MOLLI = MOdified Look Locker Inversion recovery.

**Table S4:** Normal ranges of mapping values in healthy individuals for conventional and FBmultimap sequences

|  | FBmultimap | | Conventional mapping | |
| --- | --- | --- | --- | --- |
| Parameter | Mean ± SD (ms) | Normal range (ms) | Mean ± SD (ms) | Normal range (ms) |
| T1 | 1217.6 ± 45.6 | 1126.4 to 1308.8 | 1176.7 ± 43.6 | 1089.5 to 1263.9 |
| T2 | 41.9 ± 2.7 | 36.5 to 47.3 | 42.4 ± 2.7 | 37.0 to 47.8 |
| T1ρ | 44.5 ± 3.5 | 37.5 to 51.5 | 52.5 ± 2.6 | 47.3 to 57.7 |

Note: FB = free-breathing, SD = standard deviation.

**Table S5:** Tissue characterization using conventional mapping techniques across different subgroups

|  | MOLLI T1 (ms) | T2-prepared bSSFP (ms) | T1ρ-prepared bSSFP (ms) |
| --- | --- | --- | --- |
| *Mapping values* |  |  |  |
| Control | 1176.7 ± 43.6 | 42.4 ± 2.7 | 52.5 ± 2.6 |
| HCM | 1219.1 ± 56.3 | 42.2 ± 3.0 | 55.5 ± 3.8 |
| DCM | 1223.3 ± 51.3 | 44.6 ± 4.7 | / |
| LGE+ | 1229.3 ± 58.8 | 43.2 ± 3.7 | 55.6 ± 4.3 |
| LGE− | 1198.5 ± 34.0 | 42.5 ± 3.8 | 54.7 ± 1.4 |
| *p-values* |  |  |  |
| HCM vs. Control | < 0.001 | 0.767 | 0.018 |
| DCM vs. Control | < 0.001 | 0.093 | / |
| LGE+ vs. Control | < 0.001 | 0.267 | 0.034 |
| LGE− vs. Control | 0.067 | 0.909 | 0.031 |
| LGE+ vs. LGE− | 0.035 | 0.474 | 0.609 |

Note: MOLLI = MOdified Look Locker Inversion recovery, bSSFP = balanced steady-state free precession, HCM = hypertrophic cardiomyopathy, DCM = dilated cardiomyopathy, LGE = late gadolinium enhancement.

**Table S6**: Diagnostic performance of FBmultimap (T1 + T1ρ) and FBmultimap (T1 + T2) for distinguishing LGE+ and LGE− patients from healthy controls

|  | Sequences | AUC | Optimal Cut-off | Sensitivity (%) | Specificity (%) | PPV (%) | NPV (%) |
| --- | --- | --- | --- | --- | --- | --- | --- |
| LGE+ | FBmultimap (T1 + T1ρ) | 0.904 | 0.66 | 86.3 | 84.4 | 89.8 | 79.4 |
|  | FBmultimap (T1 + T2) | 0.860 | 0.58 | 79.17 | 87.88 | 90.5 | 74.4 |
| LGE− | FBmultimap (T1 + T1ρ) | 0.859 | 0.49 | 73.7 | 84.4 | 73.7 | 84.4 |
|  | FBmultimap (T1 + T2) | 0.843 | 0.31 | 82.35 | 78.79 | 66.7 | 89.7 |

Note: LGE = late gadolinium enhancement, AUC = area under the curve, PPV = positive predictive value, NPV = negative predictive value, FB = free-breathing.
